# Therapeutic drug monitoring of antiepileptic drugs in the West Bank, Palestine: a 25-year retrospective cohort study from a multi-branch laboratory network

**DOI:** 10.1186/s12883-026-05103-0

**Published:** 2026-06-22

**Authors:** Ibrahim Salhi, Bashar Karmi

**Affiliations:** Medicare Laboratories, West Bank, Palestine

**Keywords:** Therapeutic drug monitoring, Antiepileptic drugs, Epilepsy, Palestine, West Bank, Carbamazepine, Phenytoin, Valproic acid, Pharmacokinetics, Population study, Retrospective cohort

## Abstract

**Background:**

Therapeutic drug monitoring (TDM) of antiepileptic drugs (AEDs) is essential for optimizing seizure control while minimizing toxicity, particularly in resource-constrained settings. Palestine’s West Bank healthcare system operates under profound geopolitical and infrastructural constraints. This study presents the first large-scale, multi-decade retrospective analysis of AED TDM patterns from a Palestinian multi-branch laboratory network.

**Methods:**

A retrospective cohort study was conducted using 9,232 individual AED serum level measurements from 5,197 unique patients recorded in the Laboratory Information System of Medicare Laboratories (33 branches, West Bank) from 2001 to 2026. Six AEDs were evaluated: carbamazepine, levetiracetam, lamotrigine, phenobarbital, phenytoin, and valproic acid. Results were classified as sub-therapeutic, therapeutic, or toxic against established reference ranges.

**Results:**

Of 9,232 measurements, 53.4% were therapeutic, 40.4% sub-therapeutic, and 6.1% toxic. Phenytoin showed the worst therapeutic control (31.0% therapeutic; median 8.4 mcg/mL). Valproic acid exhibited a 47.8% sub-therapeutic rate despite the highest test volume (*n* = 4,021). Lamotrigine had the highest toxic proportion (25.9%). Levetiracetam demonstrated the best control (88.6% therapeutic). Testing volume increased approximately 5.7-fold over the 25-year period.

**Conclusions:**

Frequent out-of-range AED levels, particularly for phenytoin and valproic acid, reflect complex pharmacokinetic and systemic challenges in the West Bank. The overall sub-therapeutic rate (40.4%) highlights the urgent need for enhanced TDM programs, improved patient adherence strategies, and strengthened pharmaceutical care infrastructure in this conflict-affected region.

## Introduction

Epilepsy is one of the most prevalent neurological disorders worldwide, affecting an estimated 50 million people across all age groups, socioeconomic strata, and geographic regions [1, 2]. Approximately 80% of individuals with epilepsy live in low- and middle-income countries, where access to consistent pharmacological management is frequently hampered by drug supply chain disruptions, limited specialist availability, financial constraints, and inadequate laboratory infrastructure [3]. Antiepileptic drugs (AEDs) remain the cornerstone of epilepsy treatment, with the primary therapeutic objective being complete or near-complete seizure suppression while maintaining an acceptable tolerability profile [4].

The pharmacological management of epilepsy is intrinsically complex [4–6]. Many AEDs are characterized by narrow therapeutic windows, highly variable inter-individual pharmacokinetic profiles, and clinically significant drug-drug interactions [5, 7, 8]. These properties create scenarios in which standard empirical dosing may result in persistent seizures due to sub-therapeutic drug exposure or, conversely, in toxicity from supratherapeutic accumulation [5, 6]. Therapeutic drug monitoring (TDM) — the systematic measurement of drug serum concentrations at defined steady-state intervals — provides an objective, evidence-based mechanism for individualizing AED dosing, assessing patient adherence, detecting pharmacokinetic abnormalities, and guiding clinical decision-making. TDM is therefore recognized as a standard of care in epilepsy management and is recommended by neurological societies and clinical pharmacology guidelines internationally [5, 6].

The pharmacokinetic diversity among AEDs is clinically substantial. Phenytoin exhibits saturable (Michaelis-Menten) zero-order kinetics at therapeutic concentrations, such that small dose increments can precipitate disproportionately large concentration increases, rendering TDM indispensable [9, 10]. Valproic acid and phenytoin are highly protein-bound, making serum levels susceptible to alterations in albumin concentration, renal function, and co-administered protein-bound medications [11]. Lamotrigine undergoes extensive hepatic glucuronidation that is markedly inhibited by valproic acid co-administration, often producing unexpectedly elevated serum concentrations in polytherapy regimens [7, 8]. Phenobarbital’s exceptionally long elimination half-life predisposes to drug accumulation, particularly in elderly patients and in those with hepatic impairment. In contrast, levetiracetam — a newer-generation AED — exhibits linear pharmacokinetics, minimal protein binding, and predominantly renal elimination without significant hepatic metabolism, resulting in a predictable and well-tolerated pharmacokinetic profile that makes it among the most controllable AEDs in clinical practice [12, 13].

Palestine’s West Bank presents a uniquely challenging healthcare environment. The region is home to approximately 3 million Palestinians and operates under a political and socioeconomic framework shaped by decades of conflict and complex governance structures [14]. The West Bank healthcare system functions through multiple providers including the Palestinian Authority Ministry of Health, United Nations Relief and Works Agency (UNRWA) facilities, non-governmental organizations, and a growing private sector [15, 16]. Coverage of chronic-disease medication, including AEDs, is fragmented: AEDs are obtained through Palestinian Ministry of Health outpatient pharmacies (formulary-restricted and free for patients registered in the chronic-disease program), through UNRWA primary-care pharmacies for refugees, through partial reimbursement under the Palestinian Health Insurance Corporation (PHIC) for the insured, and through direct out-of-pocket purchase at private community pharmacies for the uninsured or for second-line agents not on the MoH formulary [15, 16]. Out-of-pocket cost, formulary substitution between branded and generic preparations, and intermittent stock-out of specific AEDs are recognized features of the West Bank pharmaceutical environment that may influence adherence and dose continuity. Pharmaceutical supply chains are subject to periodic disruption related to movement restrictions, border controls, and procurement challenges, all of which can interfere with the continuous medication availability required for chronic neurological conditions such as epilepsy. Patient adherence is further affected by financial barriers, limited access to neurological specialist care, and geographic constraints imposed by internal movement restrictions.

Medicare Laboratories is among the largest private clinical laboratory networks in the West Bank, operating 33 geographically distributed branches across the region’s major governorates. Since the early 2000s, the network has provided AED TDM services to a broad cross-section of the Palestinian population, generating a longitudinal dataset of considerable epidemiological and clinical significance. Within the network, the records analyzed in the present study reflect outpatient AED TDM referrals from community physicians and primary-care practitioners, from hospital outpatient follow-up clinics, and from specialist neurology and pediatric services across the West Bank; samples are received as walk-in or appointment outpatient phlebotomy, with no inpatient samples included. Although individual referral indications were not captured in the Laboratory Information System, AED TDM in routine practice is ordinarily requested for a specific clinical reason — initiation of therapy or a dose change, suspected toxicity, suspected sub-therapeutic exposure, breakthrough seizures, suspected non-adherence, or follow-up of a patient with known instability — and the cohort analyzed here should be interpreted as a referred TDM population rather than the general Palestinian epilepsy population (this point is revisited in the Discussion and Limitations). To our knowledge, no prior large-scale, multi-decade study of AED TDM patterns has been published from the Palestinian Territories or from comparable conflict-affected settings in the Eastern Mediterranean region [17].

The present study aims to: (1) characterize the distribution of AED serum levels across therapeutic, sub-therapeutic, and toxic ranges in a Palestinian population over a 25-year period; (2) describe drug-specific distributions of serum concentrations and the proportion of measurements falling within, below, or above established therapeutic reference ranges; (3) describe the geographic and temporal distribution of TDM activity across the West Bank; and (4) discuss the clinical and public health implications of these findings for epilepsy management in resource-limited and conflict-affected settings.

## Methods

### Study design

This was a retrospective cohort study analyzing routinely collected clinical laboratory data. No prospective interventions were performed. The study protocol adhered to the principles of the Declaration of Helsinki and applicable data privacy regulations [18]. Patient records were de-identified prior to analysis. Ethical approval was obtained from the Institutional Review Board [IRB reference to be inserted upon submission].

### Data source and study setting

Data were extracted from the Laboratory Information System (LIS) of Medicare Laboratories, a privately operated multi-branch clinical laboratory network headquartered in the West Bank, Palestine. The network comprises 33 branches distributed across the West Bank’s major governorates, including Ramallah and Al-Bireh, Nablus, Hebron, Tulkarm, Bethlehem, Jenin, Jerusalem, Jericho, and Tubas. Medicare Laboratories serves both referred outpatients and inpatients from multiple healthcare facilities across the region.

### Study period

Records spanning 25 years from 2001 to 2026 were reviewed, with the predominant volume of TDM activity concentrated between 2007 and 2025. The extended timeframe enabled longitudinal assessment of evolving TDM utilization patterns and changing prescribing practices across the West Bank.

### Study population

All patients with at least one AED serum level measurement recorded in the LIS during the study period were eligible for inclusion. There were no exclusion criteria based on age, sex, indication, or comorbidity, ensuring that the dataset is representative of the real-world clinical population utilizing AED TDM services in the West Bank. The study population comprised 5,197 unique patients contributing a total of 8,775 test records and 9,232 individual drug level measurements (some records contained measurements for more than one drug).

### Antiepileptic drugs analyzed

Six AEDs were included in the analysis, representing the full spectrum of agents tested by Medicare Laboratories during the study period: carbamazepine, levetiracetam, lamotrigine, phenobarbital, phenytoin, and valproic acid. Multiple branded and generic formulations of these agents were dispensed across the West Bank during the study period; the specific brand or generic formulation administered to each patient could not be ascertained from the Laboratory Information System, and bioequivalence across formulations of narrow-therapeutic-index agents is not assured [19]. These drugs collectively represent both first-generation (phenobarbital, phenytoin, carbamazepine, valproic acid) and second-generation (lamotrigine, levetiracetam) AEDs and reflect the treatment landscape in the West Bank.

### Therapeutic reference ranges

Serum levels were classified according to established reference ranges widely adopted in clinical pharmacology practice and consistent with international guidelines [5, 6]. Results were categorized as: (1) sub-therapeutic, when serum concentrations fell below the lower limit of the therapeutic range; (2) therapeutic, when concentrations fell within the defined therapeutic window; and (3) toxic, when concentrations exceeded the upper limit of the therapeutic range. The reference ranges applied were as follows: carbamazepine 4–12 mcg/mL; levetiracetam 12–46 mcg/mL; lamotrigine 3–14 mcg/mL; phenobarbital 10–40 mcg/mL; phenytoin 10–20 mcg/mL; and valproic acid 50–100 mcg/mL.

### Demographic variables

Demographic data extracted for each patient included age at time of testing and biological sex. Age was analyzed as a continuous variable and as a categorical variable (pediatric: age < 18 years; adult: 18–64 years; elderly: >= 65 years). Geographic variables included the locality (city or village) from which the patient was referred, enabling geospatial analysis of TDM utilization across the West Bank governorates.

### Analytical methods

AED serum concentrations were measured by immunoassay methods on automated clinical chemistry analyzers using commercially validated reagent systems throughout the study period. Sampling time relative to the previous dose was not systematically recorded in the Laboratory Information System during the study period; accordingly, trough or steady-state status cannot be confirmed on a per-sample basis. Standard practice in the network was for samples to be collected immediately prior to the next scheduled dose, but adherence to this practice could not be verified for individual measurements, and an unknown fraction of below-range results may therefore reflect sampling timing rather than under-exposure. AED immunoassays were performed on Abbott platforms: prior to 2012 on the AxSym, and from 2012 onward on the ARCHITECT ci4100, using the manufacturer’s validated reagent kits for each AED. Branch-level allocation of these two platforms during the transition period (and minor reagent-generation changes within each platform) cannot be reconstructed uniformly from the LIS records. Phenytoin measurements report total phenytoin concentrations; free phenytoin and serum albumin were not available within the LIS dataset, which limits the interpretation of total-phenytoin values particularly in hypoalbuminaemic subgroups (the albumin-adjusted (Sheiner–Tozer) approach could not be applied retrospectively). Laboratory quality control protocols including internal quality control (IQC) and participation in external quality assessment (EQA) schemes were maintained throughout the study period, consistent with accreditation requirements for private medical laboratories in Palestine.

### Statistical analysis

Descriptive statistics were used to characterize the study population and the distribution of AED serum levels. Continuous variables are reported as mean with standard deviation (SD) or as median with interquartile range (IQR), depending on distributional normality. Categorical variables are reported as absolute counts and percentages. Drug-level classification (sub-therapeutic, therapeutic, toxic) was tabulated for each AED individually and for the overall dataset. The proportional distribution of classification categories across AEDs was compared descriptively. Temporal trends in TDM volume were analyzed across annual intervals. Geographic distribution of test volume was analyzed by locality and presented as both absolute counts and proportions of total testing volume. All analyses were performed using standard statistical computing software.

## Results

### Study population characteristics

A total of 9,232 AED serum level measurements from 5,197 unique patients were included in the analysis, encompassing 8,775 test records from 33 laboratory branches across the West Bank. The population was predominantly male (4,876 patients; 55.6%), with females representing 44.4% (3,899 patients). The age distribution was broad, ranging from 1 year to 119 years, reflecting the full spectrum of the population from pediatric to elderly patients. The mean age was 29.1 years (SD not available from LIS aggregate output) with a median of 24.0 years, indicating a relatively young population with a right-skewed age distribution. This demographic profile is consistent with epilepsy’s typical age of onset distribution, with disproportionate representation in childhood and early adulthood.

### Geographic distribution of TDM activity

TDM requests were distributed across all major West Bank governorates, reflecting the geographic reach of the Medicare Laboratories network. Ramallah and Al-Bireh represented the largest referral center, accounting for 3,720 measurements (40.3% of total test volume), consistent with Ramallah’s role as the administrative and tertiary-care hub of the West Bank. Nablus, Hebron, and Tulkarm followed (1,811, 1,267, and 913 measurements, respectively), and the remaining seven cities - Jenin, Bethlehem, Jerusalem, Jericho, Salfit, Tubas, and Qalqilya - together accounted for 1,521 measurements. Locality values that were unrecorded in the LIS during the data-extraction step were resolved by branch reference, allowing every measurement (*n* = 9,232) in the cohort to be allocated to a city.

### Temporal trends in TDM Utilization

**Fig. 1:**
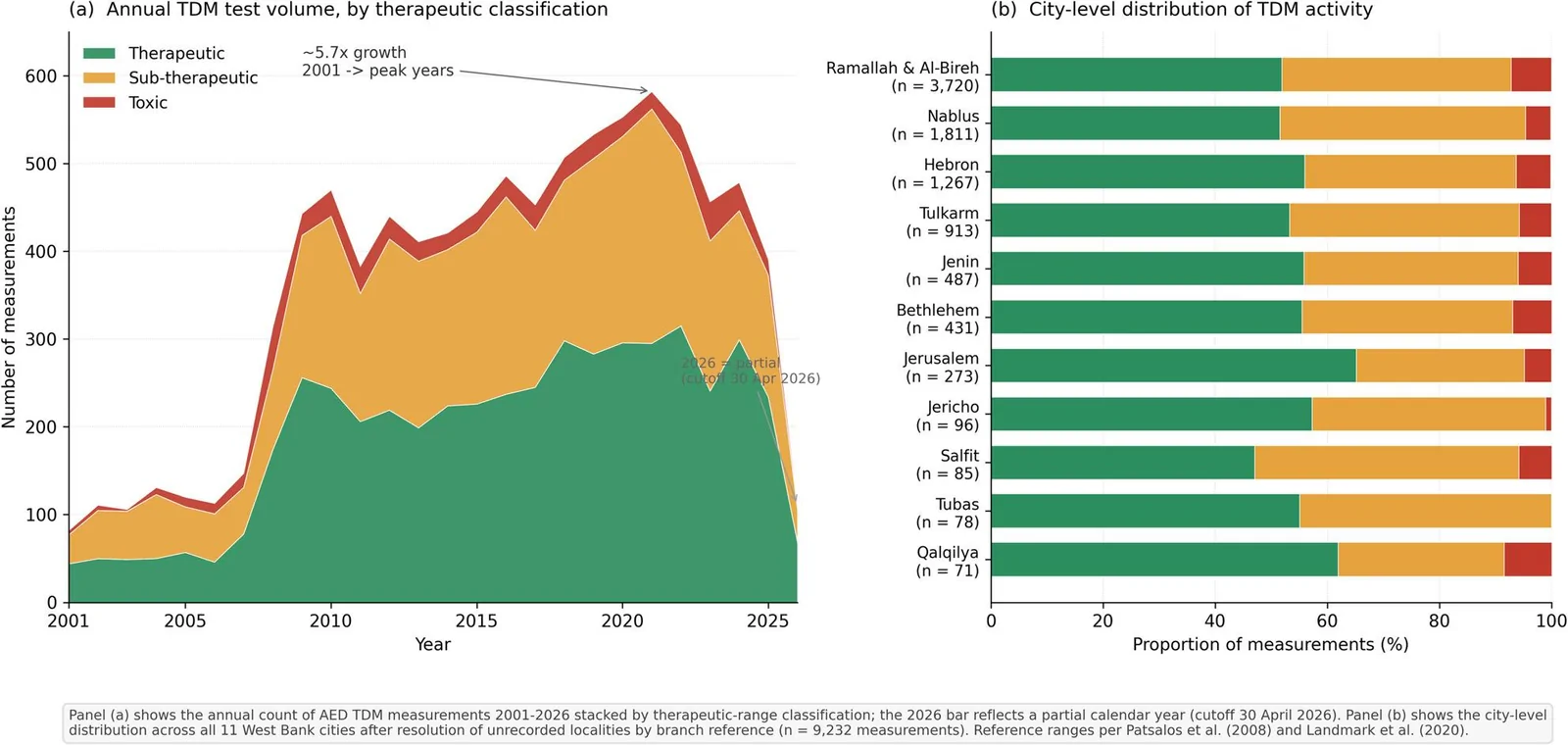
Temporal growth and city-level distribution of AED TDM activity, Medicare Laboratories network, West Bank (2001–2026). (*N* = 9,232 measurements from 5,197 patients across 33 branches). Panel (**a**) shows the annual count of AED TDM measurements 2001–2026 stacked by therapeutic-range classification; the 2026 bar reflects a partial calendar year (cutoff 30 April 2026). Panel (**b**) shows the city-level distribution of measurements aggregated across all Medicare Laboratories branches serving each city (*n* = 8,671 measurements with resolvable city of locality; 561 measurements without resolvable city are excluded from panel b but retained in the overall denominator of 9,232 used in Tables 1 and 2)

TDM activity demonstrated a significant upward trajectory over the 25-year study period. Annual test volumes increased approximately 5.7-fold from 2001 to recent years, with the period 2007–2025 accounting for the majority of measurements. This growth pattern reflects the progressive development of TDM infrastructure within Palestinian laboratory medicine, expansion of the Medicare Laboratories branch network, and increasing awareness among prescribers of the clinical utility of serum drug level monitoring in AED management. Annual TDM test volumes by therapeutic classification and the city-level distribution of measurements are shown in Fig. 1.

### Overall therapeutic level classification

**Fig. 2:**
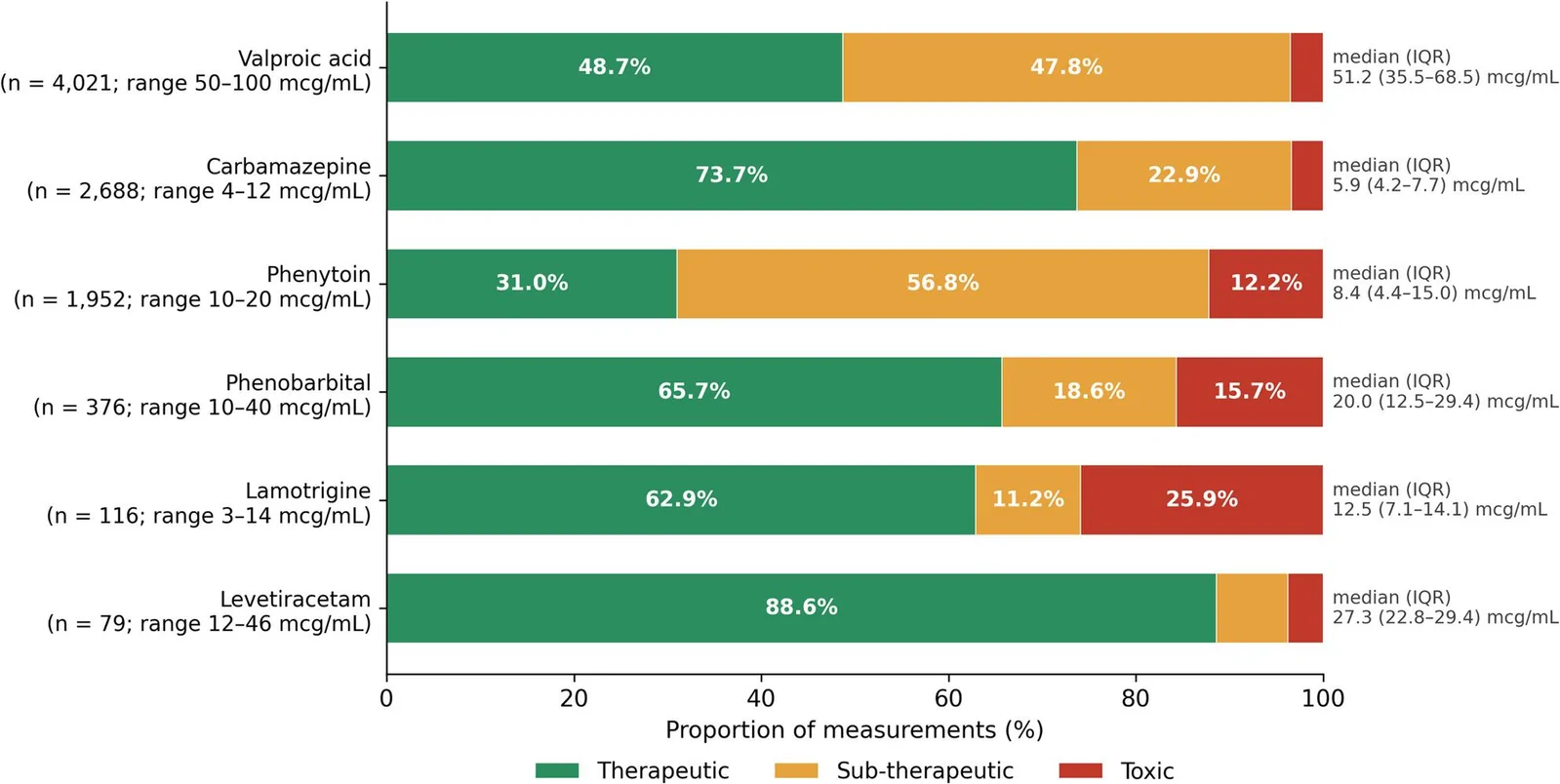
Drug-specific therapeutic-range classification of serum AED measurements (Medicare Laboratories network, West Bank, 2001–2026). Therapeutic-range classification of serum AED measurements, by drugMedicare Laboratories network, West Bank (2001–2026; *N* = 9,232 measurements from 5,197 patients). Figure 1 displays the proportion of measurements in the therapeutic, sub-therapeutic, and toxic categories for each of the six AEDs, with sample size, reference range, and median (IQR) annotated. Reference ranges per Patsalos et al. (2008) and Landmark et al. (2020). Total-phenytoin concentrations only; free phenytoin and serum albumin were not available. Sampling time relative to dose was not recorded

Across all six AEDs combined, 4,933 measurements (53.4%) fell within the therapeutic range, 3,734 (40.4%) were sub-therapeutic, and 565 (6.1%) were toxic. These findings indicate that nearly half of all AED measurements obtained in clinical practice in the West Bank fell outside the target therapeutic window, with the sub-therapeutic category predominating. Table 1 summarizes drug-specific results. The drug-specific therapeutic-range distribution is shown in Fig. 2 (Tables 2 and 3).

**Table 1:** Summary of antiepileptic drug serum level classifications in the west bank (2001–2026)

Drug	Therapeutic range (mcg/mL)	*N* tests	Therapeutic *n* (%)	Sub-therapeutic *n* (%)	Toxic *n* (%)	Median (mcg/mL)	IQR (mcg/mL)
Valproic acid	50–100	4,021	1,957 (48.7%)	1,922 (47.8%)	142 (3.5%)	51.2	35.5–68.5
Carbamazepine	4–12	2,688	1,981 (73.7%)	615 (22.9%)	92 (3.4%)	5.9	4.2–7.7
Phenytoin	10–20	1,952	605 (31.0%)	1,108 (56.8%)	239 (12.2%)	8.4	4.4–15.0
Phenobarbital	10–40	376	247 (65.7%)	70 (18.6%)	59 (15.7%)	20.0	12.5–29.4
Lamotrigine	3–14	116	73 (62.9%)	13 (11.2%)	30 (25.9%)	12.5	7.1–14.1
Levetiracetam	12–46	79	70 (88.6%)	6 (7.6%)	3 (3.8%)	27.3	22.8–29.4
TOTAL	**—**	9,232	4,933 (53.4%)	3,734 (40.4%)	565 (6.1%)	**—**	**—**

Drugs are listed in descending order of test volume. Therapeutic, sub-therapeutic, and toxic categories are defined relative to the reference ranges shown in column 2 (Patsalos et al., 2008; Landmark et al., 2020). IQR = interquartile range (25th–75th percentiles). Brand names previously included in this table (Tegretol, Keppra, Lamictal, Luminal, Epanutin, Depalept) have been removed because the specific brand or generic formulation administered to each patient could not be ascertained from the Laboratory Information System; bioequivalence across formulations of narrow-therapeutic-index agents is not assured. The asterisk and dagger symbols previously footnoted with the phenytoin and valproic acid medians (free-fraction caveat and enzyme-induction caveat, respectively) have been replaced by an explicit note: total — not free — phenytoin was measured and albumin was unavailable, which limits interpretation of the phenytoin median; the valproic acid median should be interpreted alongside the recognized intra-individual diurnal variation in valproate levels and the high proportion of below-range results

**Table 2:** Annual TDM test volume and therapeutic classification (2001–2026)

Year	*N* Tests	Therapeutic *n* (%)	Sub-therapeutic *n* (%)	Toxic *n* (%)
2001	82	44 (53.7%)	33 (40.2%)	5 (6.1%)
2002	111	50 (45.0%)	55 (49.5%)	6 (5.4%)
2003	106	49 (46.2%)	55 (51.9%)	2 (1.9%)
2004	131	50 (38.2%)	73 (55.7%)	8 (6.1%)
2005	120	57 (47.5%)	52 (43.3%)	11 (9.2%)
2006	113	46 (40.7%)	55 (48.7%)	12 (10.6%)
2007	147	78 (53.1%)	53 (36.1%)	16 (10.9%)
2008	314	174 (55.4%)	91 (29.0%)	49 (15.6%)
2009	443	256 (57.8%)	162 (36.6%)	25 (5.6%)
2010	470	244 (51.9%)	196 (41.7%)	30 (6.4%)
2011	383	206 (53.8%)	146 (38.1%)	31 (8.1%)
2012	440	219 (49.8%)	195 (44.3%)	26 (5.9%)
2013	411	199 (48.4%)	190 (46.2%)	22 (5.4%)
2014	421	224 (53.2%)	178 (42.3%)	19 (4.5%)
2015	445	226 (50.8%)	196 (44.0%)	23 (5.2%)
2016	486	237 (48.8%)	225 (46.3%)	24 (4.9%)
2017	453	245 (54.1%)	179 (39.5%)	29 (6.4%)
2018	507	298 (58.8%)	183 (36.1%)	26 (5.1%)
2019	533	283 (53.1%)	223 (41.8%)	27 (5.1%)
2020	553	296 (53.5%)	235 (42.5%)	22 (4.0%)
2021	582	295 (50.7%)	267 (45.9%)	20 (3.4%)
2022	544	315 (57.9%)	198 (36.4%)	31 (5.7%)
2023	457	241 (52.7%)	171 (37.4%)	45 (9.8%)
2024	478	299 (62.6%)	147 (30.8%)	32 (6.7%)
2025	391	234 (59.8%)	139 (35.5%)	18 (4.6%)
2026	111	68 (61.3%)	37 (33.3%)	6 (5.4%)
TOTAL	9232	4933 (53.4%)	3734 (40.4%)	565 (6.1%)

The 2026 row reflects a partial calendar year; data were extracted up to the cutoff date of 30 April 2026. Therapeutic, sub-therapeutic, and toxic categories are defined drug-by-drug relative to the reference ranges shown in Table 1

**Table 3:** Geographic distribution of TDM activity by laboratory branch (2001–2026)

City	*N* tests	% of total	Therapeutic *n* (%)	Sub-therapeutic *n* (%)	Toxic *n* (%)
Ramallah and Al-Bireh	3,720	40.3%	1,930 (51.9%)	1,522 (40.9%)	268 (7.2%)
Nablus	1,811	19.6%	935 (51.6%)	794 (43.8%)	82 (4.5%)
Hebron	1,267	13.7%	710 (56.0%)	478 (37.7%)	79 (6.2%)
Tulkarm	913	9.9%	487 (53.3%)	374 (41.0%)	52 (5.7%)
Jenin	487	5.3%	272 (55.9%)	186 (38.2%)	29 (6.0%)
Bethlehem	431	4.7%	239 (55.5%)	162 (37.6%)	30 (7.0%)
Jerusalem	273	3.0%	178 (65.2%)	82 (30.0%)	13 (4.8%)
Jericho	96	1.0%	55 (57.3%)	40 (41.7%)	1 (1.0%)
Salfit	85	0.9%	40 (47.1%)	40 (47.1%)	5 (5.9%)
Tubas	78	0.8%	43 (55.1%)	35 (44.9%)	0 (0.0%)
Qalqilya	71	0.8%	44 (62.0%)	21 (29.6%)	6 (8.5%)
TOTAL	9,232	100.0%	4,933 (53.4%)	3,734 (40.4%)	565 (6.1%)

The geographic distribution is presented at the city level rather than at the level of individual branches; rows aggregate all Medicare Laboratories branches serving each city. Locality values that were unrecorded in the Laboratory Information System during the data-extraction step were subsequently resolved by branch reference, and all 9,232 measurements are allocated to a city. Below-detection (< limit) and above-limit (> limit) results are counted as sub-therapeutic and toxic, respectively, consistent with the drug-summary classification in Table 1

### Drug-specific findings

#### Carbamazepine

Carbamazepine demonstrated the highest therapeutic attainment rate among the frequently tested AEDs, with 1,981 of 2,688 measurements (73.7%) falling within the target range of 4–12 mcg/mL. The sub-therapeutic rate was 22.9% (615 measurements) and the toxic rate was 3.4% (92 measurements). The median serum concentration was 5.9 mcg/mL. These results reflect carbamazepine’s well-established pharmacokinetic profile and the relative ease of dose titration in clinical practice, although the 22.9% sub-therapeutic rate still represents a clinically significant proportion of uncontrolled or under-treated patients.

#### Levetiracetam

Levetiracetam achieved the highest therapeutic attainment rate of all six AEDs studied, with 70 of 79 measurements (88.6%) within the therapeutic range of 12–46 mcg/mL. Only 6 measurements (7.6%) were sub-therapeutic and 3 (3.8%) were toxic. The median concentration was 27.3 mcg/mL. Despite the small sample size relative to other AEDs, these results are consistent with levetiracetam’s predictable pharmacokinetics and manageable drug-interaction profile. Given levetiracetam’s linear pharmacokinetics, minimal protein binding, and absence of clinically significant CYP-mediated interactions, routine TDM for levetiracetam is not generally indicated outside specific clinical scenarios such as renal impairment, pregnancy, suspected non-adherence, suspected toxicity, or extremes of body weight; the small number of levetiracetam measurements in this cohort is consistent with appropriately selective use of the assay [5, 20].

#### Lamotrigine

Lamotrigine results revealed a striking pattern of toxicity, with 30 of 116 measurements (25.9%) exceeding the upper therapeutic limit of 14 mcg/mL — the highest toxic rate of all AEDs in this study. The therapeutic attainment rate was 62.9% (73 measurements) and the sub-therapeutic rate was 11.2% (13 measurements). The median concentration was 12.5 mcg/mL, which is near the upper boundary of the therapeutic range, suggesting a systematic tendency toward elevated concentrations in this population. This pattern is consistent with pharmacokinetic interactions involving co-prescribed valproic acid, which inhibits lamotrigine glucuronidation and can double or triple lamotrigine serum concentrations.

#### Phenobarbital

Phenobarbital showed a therapeutic attainment rate of 65.7% (247 of 376 measurements within the 10–40 mcg/mL reference range), a sub-therapeutic rate of 18.6% (70 measurements), and a toxic rate of 15.7% (59 measurements). The median concentration was 20.0 mcg/mL. The elevated toxic rate — the second highest among all AEDs — is consistent with phenobarbital’s long elimination half-life (typically 96–120 h in adults), which predisposes to drug accumulation, particularly in elderly patients and those with hepatic or renal impairment. The 15.7% toxicity rate suggests that phenobarbital dosing intervals and monitoring frequency may require recalibration in the West Bank patient population.

#### Phenytoin

Phenytoin exhibited the lowest therapeutic attainment rate of all AEDs, with only 605 of 1,952 measurements (31.0%) within the target range of 10–20 mcg/mL. Sub-therapeutic levels predominated, accounting for 1,108 measurements (56.8%), while 239 measurements (12.2%) were toxic. The median serum concentration was 8.4 mcg/mL — well below the lower therapeutic boundary — confirming a systematic pattern of under-treatment. Notably, the median is marked with an asterisk (*) indicating that the reported level likely underestimates pharmacologically active free phenytoin concentrations in patients with hypoalbuminemia, as total phenytoin binding is approximately 90% protein-bound. When albumin levels are reduced — common in the chronically ill or malnourished — free phenytoin fractions increase substantially, meaning that some levels classified as sub-therapeutic on total-phenytoin assay may in fact represent adequate or even toxic free-drug exposure.

#### Valproic acid

Valproic acid accounted for the largest share of AED testing volume, with 4,021 measurements representing 43.6% of the total dataset. Despite this high monitoring frequency, therapeutic attainment was achieved in only 1,957 measurements (48.7%), with sub-therapeutic levels in 1,922 measurements (47.8%) and toxic levels in 142 measurements (3.5%). The median concentration was 51.2 mcg/mL, marked with a dagger (†) indicating that this value must be interpreted in the context of valproate’s saturable protein binding: at concentrations above approximately 75–80 mcg/mL, protein binding sites become saturated and the free fraction increases non-linearly, meaning total VPA assays may underestimate pharmacodynamic exposure at higher concentrations. The near-equal split between therapeutic and sub-therapeutic results, combined with the high testing frequency, suggests that valproic acid is being monitored appropriately but that dose optimisation remains challenging in clinical practice.

### Therapeutic-range classification by sex and age group

The overall classification of measurements by sex and by age group is shown in Table 4. Female-derived samples were more frequently within range (56.8%) than male-derived samples (50.8%); pediatric measurements (< 18 years) and adult measurements (18–64 years) showed broadly similar therapeutic attainment rates (53.9% and 54.2%, respectively), while measurements from elderly patients ( > = 65 years) showed the lowest therapeutic attainment (46.9%) and an essentially balanced split between therapeutic and sub-therapeutic (46.3%) classifications. These descriptive subgroup proportions are reported as a feature of the dataset and are not adjusted for drug, indication, or other covariates.

**Table 4:** Therapeutic-range classification of AED serum levels by sex and age group (2001–2026)

Stratum	Subgroup	*N* tests	Therapeutic *n* (%)	Sub-therapeutic *n* (%)	Toxic *n* (%)
Sex	Female	4,069	2,310 (56.8%)	1,490 (36.6%)	269 (6.6%)
	Male	5,163	2,623 (50.8%)	2,244 (43.5%)	296 (5.7%)
Age group	Pediatric (< 18 y)	3,015	1,624 (53.9%)	1,240 (41.1%)	151 (5.0%)
	Adult (18–64 y)	5,427	2,939 (54.2%)	2,126 (39.2%)	362 (6.7%)
	Elderly ( > = 65 y)	657	308 (46.9%)	304 (46.3%)	45 (6.8%)
	Unknown age	133	62 (46.6%)	64 (48.1%)	7 (5.3%)
Total (all strata)	**-**	9,232	4,933 (53.4%)	3,734 (40.4%)	565 (6.1%)

Stratification was performed at the individual-measurement level (one row per serum AED measurement). Below-detection and above-limit results are counted as sub-therapeutic and toxic, respectively, consistent with Table 1. The Unknown-age subgroup captures 133 measurements for which age was not recorded; sex was available for all 9,232 measurements after data resolution. Percentages are row percentages within each subgroup

## Discussion

This retrospective cohort study presents the largest and most comprehensive analysis of AED TDM data published from Palestine or, to our knowledge, from any conflict-affected setting in the Eastern Mediterranean region. The 25-year dataset, encompassing 9,232 measurements from 5,197 patients across 33 laboratory branches, provides unique insights into real-world AED TDM activity in a resource-constrained population. Before discussing drug-specific findings, the referral context of the dataset should be emphasized. TDM of AEDs is ordinarily requested for a specific clinical reason — initiation of therapy or a dose change, suspected toxicity, suspected sub-therapeutic exposure, breakthrough seizures, or follow-up in a patient with known instability — and the proportion of results falling outside the therapeutic reference range therefore reflects the indication for testing at least as much as the underlying quality of epilepsy control in the population. The out-of-range proportions reported in this dataset should accordingly be interpreted as a description of TDM activity and of the laboratory signal seen by the West Bank TDM service, not as a direct index of population-level epilepsy control quality, and the discussion that follows is framed in those terms. Our central finding — that 53.4% of all AED measurements fell within established therapeutic ranges, while 40.4% were sub-therapeutic and 6.1% toxic — has implications for epilepsy management policy, clinical practice, and laboratory medicine development in Palestine.

### Phenytoin: lowest therapeutic attainment rate and candidate explanations

The lowest therapeutic attainment rate observed in this study was for phenytoin: 31.0% of measurements within the conventional reference range, 56.8% sub-therapeutic, and a median total-phenytoin serum level of 8.4 mcg/mL — 16% below the lower therapeutic limit. Candidate explanations for this finding — none of which can be tested with the variables captured in this dataset — include free-fraction effects under hypoalbuminaemia (only total, not free, phenytoin was measured, and albumin was not available in the LIS), sampling time relative to dose, adherence patterns, dose at the time of sampling, and supply-related dose interruptions. We do not infer systematic under-treatment from these data. Phenytoin’s challenging pharmacokinetics are central to interpreting these results as a general mechanism. At therapeutic concentrations, phenytoin exhibits saturable, zero-order (Michaelis-Menten) kinetics: as dose increases within the therapeutic range, hepatic metabolic capacity becomes progressively saturated, producing disproportionately large serum concentration increases relative to dose increments. Conversely, small reductions in dose — or missed doses — can produce disproportionate concentration decreases, rapidly pushing levels into the sub-therapeutic range.

Several additional pharmacokinetic factors compound phenytoin’s management complexity in the Palestinian context. First, phenytoin is approximately 90% bound to serum albumin, and the free (pharmacologically active) fraction increases substantially in states of hypoalbuminemia, renal insufficiency, or concurrent administration of other protein-bound drugs. Standard total phenytoin measurements may therefore significantly underestimate free drug exposure in patients with hypoalbuminemia — a condition that may be prevalent in a population where nutritional and socioeconomic challenges are common. Because albumin was not available in the LIS and the assay measured total rather than free phenytoin, the Sheiner–Tozer albumin-adjusted approach could not be applied retrospectively in this dataset; this is acknowledged as a limitation of the assay used (Sect. 2.8) [21, 22]. Second, phenytoin is a potent inducer of cytochrome P450 (CYP) enzymes, particularly CYP2C9 and CYP3A4, creating complex drug-drug interaction potentials with co-prescribed medications that may alter its own metabolism [7, 8]. Third, the narrow therapeutic window (10–20 mcg/mL) combined with non-linear kinetics creates a particularly steep dose-response curve that demands precision dosing and frequent monitoring [9]. Because individual doses, dosing intervals, sampling times, comedications, and albumin status were not captured in this dataset, the relative contribution of empirical under-dosing, free-fraction effects, sampling timing, adherence, and supply interruptions to the high sub-therapeutic prevalence observed cannot be determined; these candidate explanations are offered as hypotheses that the present descriptive dataset cannot test [5, 6].

### Valproic acid: the paradox of high testing volume and low therapeutic attainment

Valproic acid represented 43.6% of all AED measurements in this study, making it by far the most monitored drug in the cohort — yet nearly half of all measurements (47.8%) were sub-therapeutic. This paradox of high TDM utilization combined with poor therapeutic control underscores the inherent pharmacokinetic challenges of valproic acid management. Valproic acid is highly protein-bound (approximately 90% at therapeutic concentrations), with free fraction increasing non-linearly as total drug concentrations rise — a property that can lead to discrepancies between total measured concentrations (as captured in this dataset) and pharmacologically active free concentrations [11, 23].

Drug-drug interactions are a recognized contributor to valproic acid sub-therapeutic levels in general clinical practice. Enzyme-inducing AEDs — notably carbamazepine — markedly increase valproic acid clearance through induction of CYP2C9 and beta-oxidation pathways, reducing serum concentrations by up to 50% in polytherapy regimens [7, 8, 24]. Because comedication, dose, and patient-level linkage between valproate and carbamazepine tests were not available in this dataset, we cannot establish the prevalence of valproate–carbamazepine co-prescription in this cohort; this is offered as a recognized pharmacological mechanism that may contribute, framed as a hypothesis that the present data cannot test. Patient non-adherence is an additional factor relevant in general clinical practice: valproic acid’s well-documented side effect profile (including weight gain, hair loss, teratogenicity concerns in women of childbearing age, and tremor) is associated with reduced long-term adherence [25]. Medication cost and intermittent drug availability — features of the Palestinian pharmaceutical supply chain — are plausible additional contributors at the population level. The predominance of the sub-therapeutic category over the toxic category (47.8% vs. 3.5%) is consistent with under-exposure rather than toxicity being the predominant signal in the valproate measurements observed here; the dataset does not permit attribution of this signal to any one mechanism.

### Lamotrigine toxicity: the valproic acid interaction as a population-level pharmacokinetic signal

The 25.9% toxic rate for lamotrigine in this cohort is substantially higher than rates reported in general epilepsy populations receiving lamotrigine monotherapy and represents the highest toxic proportion of any AED in this study. The lamotrigine–valproate interaction at UDP-glucuronosyltransferase 1A4 (UGT1A4) is a well-characterized pharmacological mechanism in which valproic acid inhibits the primary glucuronidation pathway for lamotrigine [7, 26]. Co-administration of valproic acid at standard doses can double or triple lamotrigine serum concentrations, producing levels within or above the toxic range at doses that would be sub-therapeutic in the absence of valproic acid [8, 24]. Because this dataset does not record comedications, prescribed dose, or patient-level linkage between lamotrigine and valproic acid measurements, we cannot establish whether the two drugs are being co-prescribed in this cohort, and we therefore cannot attribute the observed lamotrigine toxicity rate to a population-level prescribing pattern. The lamotrigine/valproate UGT1A4 interaction is offered here only as a recognized pharmacological mechanism that may contribute to lamotrigine concentrations in individual patients who happen to be receiving both drugs; this remains a hypothesis that the present data cannot test. As general clinical guidance independent of this dataset, lamotrigine doses in patients receiving concurrent valproic acid should typically be reduced to 25–50% of standard doses, with more frequent TDM at the time of combination therapy.

### Phenobarbital: accumulation risk in an aging cohort

The 15.7% toxic rate for phenobarbital — the second highest of all AEDs in this cohort — is attributable primarily to the drug’s pharmacokinetic properties. Phenobarbital’s exceptionally long elimination half-life (80–100 h in healthy adults, extending to 100–150 h in elderly patients and those with hepatic impairment) creates substantial accumulation risk, particularly when dose adjustments are made without adequate washout periods between increments [9, 10]. A dose adjustment that appears clinically appropriate at the time of administration may produce toxicity 2–3 weeks later as accumulation occurs across multiple half-lives to reach a new steady state. The relatively wide therapeutic range (10–40 mcg/mL) does not mitigate this risk in clinical practice, as toxic manifestations (sedation, ataxia, cognitive impairment) can occur within the upper portion of the range in sensitive individuals. The 15.7% toxic proportion in this cohort indicates that phenobarbital accumulation is a clinically prevalent phenomenon in the West Bank population.

### Levetiracetam: a model of pharmacokinetic predictability

The 88.6% therapeutic attainment rate for levetiracetam stands in stark contrast to the other AEDs in this study and reflects the drug’s uniquely favorable pharmacokinetic profile. Levetiracetam is minimally protein-bound (less than 10%), undergoes renal elimination via hydrolysis without significant hepatic CYP metabolism, and exhibits linear pharmacokinetics across the dosing range [12, 13]. Consequently, it does not participate in the CYP-mediated drug-drug interactions that complicate the management of carbamazepine, phenytoin, valproic acid, and lamotrigine [24, 26]. These properties produce a dose-concentration relationship that is predictable, consistent, and largely unaffected by comorbidities (except renal impairment), co-medications, or fluctuations in nutritional status. The levetiracetam data from this cohort therefore serves as a pharmacokinetic benchmark: the 88.6% therapeutic attainment rate achieved with minimal dose complexity illustrates the degree to which poor therapeutic control with other AEDs is pharmacokinetically driven rather than reflecting failures of TDM practice per se.

### Systemic contextual factors: the west bank healthcare environment

The overall sub-therapeutic rate of 40.4% across all AEDs cannot be explained by pharmacokinetic mechanisms alone. Contextual factors specific to the West Bank healthcare environment are likely major contributors to this finding [14, 17]. Medication non-adherence is a universal challenge in chronic disease management but is amplified in the West Bank by several specific factors [3]. Financial barriers affect a significant proportion of the population, particularly those without health insurance coverage. The Palestinian pharmaceutical market is subject to supply chain disruptions arising from geopolitical constraints, import restrictions, and logistical challenges associated with operating under occupation, which can lead to medication shortages and enforced treatment interruptions [15, 16]. Limited access to neurological specialist care — neurologists are concentrated in major urban centers, and movement restrictions can limit patient access — may result in infrequent medication reviews and suboptimal dose titration.

Additionally, health literacy related to the consequences of missed doses and the necessity of adhering to twice- or three-times-daily AED regimens may be insufficient in a population with limited access to patient education resources. The geographic concentration of TDM activity in Ramallah (38.2% of all tests), which reflects its role as the administrative and healthcare hub of the West Bank, further suggests that patients in rural and peripheral areas face additional barriers to TDM access, potentially receiving less monitoring than clinically indicated.

### Temporal trends and implications for TDM program development

The 5.7-fold increase in AED TDM volume over the 25-year study period represents a significant achievement in the development of laboratory medicine infrastructure in Palestine. This growth reflects investment by Medicare Laboratories in multi-site laboratory capacity, analytical technology upgrades, and outreach to prescribers across the West Bank. However, growing test volume alone does not translate into therapeutic benefit if the clinical response to TDM results remains suboptimal. The high sub-therapeutic rates documented in this study, despite sustained growth in TDM utilization, suggest a gap between TDM measurement and clinical action — a phenomenon described in the international TDM literature as the ‘monitoring-to-action’ gap [27]. Closing this gap requires not only laboratory infrastructure but also clinician education, pharmacist-led TDM interpretation services, structured dose adjustment protocols, and integration of TDM reports into patient care pathways [6, 28, 29].

### Comparison with regional and international data

Comparative data from similar settings are limited but informative. Studies from neighboring Arab countries including Jordan, Egypt, and Lebanon have reported sub-therapeutic AED rates ranging from 30% to 55%, consistent with the 40.4% sub-therapeutic rate observed in this cohort [17, 27]. A large retrospective study from Saudi Arabia reported phenytoin therapeutic attainment rates of 35–40%, somewhat higher than the 31.0% observed here, potentially reflecting greater resource availability in that setting [30]. The particularly high lamotrigine toxicity rate (25.9%) reported here is notably higher than rates reported in most international single-center studies and likely reflects the specific polytherapy pattern of the West Bank population [26]. Studies from sub-Saharan Africa, where epilepsy management challenges share some contextual similarities with Palestine, have reported even lower therapeutic attainment rates [3], emphasizing that the West Bank, while facing significant challenges, benefits from a functioning private laboratory network providing TDM services.

### Limitations

Several limitations of this study should be acknowledged. First, as a retrospective analysis of routine LIS data, the study does not capture clinical context including indication, prescribed dose, dosing interval, time since last dose at sampling, co-medications, or seizure frequency. The absence of individual dose and sampling time data precludes pharmacokinetic modeling or assessment of sample timing appropriateness. In particular, the absence of recorded sampling time relative to dose means that an unknown fraction of below-range results may reflect sample timing rather than under-exposure, and the absence of comedication data and per-patient linkage means that no mechanistic or population-level prescribing inference (including lamotrigine–valproate co-prescription) can be drawn from the present dataset. Second, the study captures patients who underwent TDM testing and therefore does not represent the full population of patients receiving AED therapy in the West Bank. Because TDM is ordinarily requested for a specific clinical reason, the cohort is a referred TDM population rather than the general Palestinian epilepsy population, and the out-of-range proportions reported are an index of the laboratory signal in those referred for monitoring rather than a direct measure of population-level epilepsy control quality. Third, the broad age range and clinical heterogeneity of the cohort (pediatric to elderly patients, varying indications) limit the precision of drug-level interpretation across subgroups. Fourth, laboratory methodology evolved over the 25-year study period, and analytical variation across different immunoassay platforms used at different branches over time may have introduced minor inter-site and inter-period measurement variability. Fifth, data on albumin levels, renal function, and hepatic function — parameters critical for interpreting phenytoin and valproic acid levels — were not available in the LIS dataset; total — not free — phenytoin was measured, and the Sheiner–Tozer albumin adjustment could not be applied retrospectively. Sixth, while the study encompasses 33 branches, the extent to which patients from all geographic areas of the West Bank are represented equally cannot be confirmed.

## Conclusion

This 25-year retrospective analysis of 9,232 AED serum level measurements from 5,197 patients across 33 laboratory branches in the West Bank, Palestine, demonstrates that the majority of monitored patients do not achieve consistent therapeutic drug levels. Only 53.4% of all measurements were therapeutic, while 40.4% were sub-therapeutic and 6.1% toxic. These findings carry urgent clinical and policy implications.

Phenytoin showed the lowest therapeutic attainment rate: 31.0% of measurements were within the conventional reference range, with a median total-phenytoin level 16% below the lower therapeutic limit. Because total — not free — phenytoin was measured, albumin status was unavailable, and sampling time was not recorded, the relative contribution of free-fraction effects, sampling timing, adherence, and dose to this finding cannot be determined from this dataset, and we do not infer systematic under-treatment. Clinicians managing phenytoin should account for its non-linear kinetics and high protein binding, and free-phenytoin or albumin-adjusted approaches should be considered where the assay and clinical context permit. Valproic acid’s 47.8% sub-therapeutic rate — despite being the most-monitored AED in the cohort — is consistent with the combined impact of drug-drug interactions with enzyme-inducing co-AEDs, patient adherence challenges, and supply chain limitations as candidate explanations at the population level. The 25.9% lamotrigine toxic rate is consistent with the recognized lamotrigine–valproate UGT1A4-mediated interaction as one candidate mechanism but cannot be attributed to population-level co-prescription in the absence of comedication data; the dose-reduction guidance for individual patients receiving combination therapy stands as established pharmacological practice independent of these data. Levetiracetam’s 88.6% therapeutic attainment rate is consistent with its favorable pharmacokinetics, although the small sample size and the appropriately selective indications for levetiracetam TDM limit broader inference.

At the system level, the overall sub-therapeutic rate of 40.4% points to structural challenges in epilepsy management across the West Bank: inadequate patient adherence support, pharmaceutical supply chain vulnerabilities, limited access to neurological specialist care, and insufficient pharmacist integration into TDM interpretation workflows. The 5.7-fold growth in TDM volume over 25 years demonstrates the expanding capacity of Palestinian laboratory medicine but highlights the need to translate measurement volume into clinical action.

We recommend the following priority interventions: (1) implementation of albumin-adjusted phenytoin monitoring protocols across all West Bank TDM facilities; (2) mandatory lamotrigine dose reduction protocols for patients initiating concurrent valproic acid therapy; (3) development of pharmacist-led TDM interpretation and clinical consultation services; (4) patient adherence support programs tailored to the West Bank context, including community-based pharmacy engagement; (5) investment in decentralized TDM capacity to reduce the geographic concentration of services in Ramallah and improve access in peripheral governorates; and (6) establishment of a prospective AED TDM registry to enable longitudinal outcome tracking. This study establishes a critical evidence base for these interventions and provides a reference dataset against which future improvements in epilepsy care quality can be measured.

## Data Availability

The datasets generated and analyzed during the current study are not publicly available because they contain pseudonymized individual patient laboratory data governed by Medicare Laboratories institutional data-protection policies and by the Palestinian Ministry of Health regulations on patient health information, but are available in de-identified form from the corresponding author (Ibrahim Salhi, gmd@medicare.ps) on reasonable request and subject to approval by the Medicare Laboratories Institutional Review Board.
